# Molecular Characterization of Human T-Cell Lymphotropic Virus Type 1 Full and Partial Genomes by Illumina Massively Parallel Sequencing Technology

**DOI:** 10.1371/journal.pone.0093374

**Published:** 2014-03-31

**Authors:** Rodrigo Pessôa, Jaqueline Tomoko Watanabe, Youko Nukui, Juliana Pereira, Jorge Kasseb, Augusto César Penalva de Oliveira, Aluisio Cotrim Segurado, Sabri Saeed Sanabani

**Affiliations:** 1 Department of Virology, São Paulo Institute of Tropical Medicine, São Paulo, Brazil; 2 Department of Hematology, Faculty of Medicine, University of São Paulo, São Paulo, Brazil; 3 Department of Neurology, Institute of Infectology Emilio Ribas, São Paulo, Brazil; 4 Department of Infectious Diseases, Faculty of Medicine, University of São Paulo, São Paulo, Brazil; 5 Clinical Laboratory, Department of Pathology, Hospital das Clínicas, School of Medicine, University of São Paulo, São Paulo, Brazil; Centers for Disease Control and Prevention, United States of America

## Abstract

**Background:**

Here, we report on the partial and full-length genomic (FLG) variability of HTLV-1 sequences from 90 well-characterized subjects, including 48 HTLV-1 asymptomatic carriers (ACs), 35 HTLV-1-associated myelopathy/tropical spastic paraparesis (HAM/TSP) and 7 adult T-cell leukemia/lymphoma (ATLL) patients, using an Illumina paired-end protocol.

**Methods:**

Blood samples were collected from 90 individuals, and DNA was extracted from the PBMCs to measure the proviral load and to amplify the HTLV-1 FLG from two overlapping fragments. The amplified PCR products were subjected to deep sequencing. The sequencing data were assembled, aligned, and mapped against the HTLV-1 genome with sufficient genetic resemblance and utilized for further phylogenetic analysis.

**Results:**

A high-throughput sequencing-by-synthesis instrument was used to obtain an average of 3210- and 5200-fold coverage of the partial (n = 14) and FLG (n = 76) data from the HTLV-1 strains, respectively. The results based on the phylogenetic trees of consensus sequences from partial and FLGs revealed that 86 (95.5%) individuals were infected with the transcontinental sub-subtypes of the cosmopolitan subtype (aA) and that 4 individuals (4.5%) were infected with the Japanese sub-subtypes (aB). A comparison of the nucleotide and amino acids of the FLG between the three clinical settings yielded no correlation between the sequenced genotype and clinical outcomes. The evolutionary relationships among the HTLV sequences were inferred from nucleotide sequence, and the results are consistent with the hypothesis that there were multiple introductions of the transcontinental subtype in Brazil.

**Conclusions:**

This study has increased the number of subtype aA full-length genomes from 8 to 81 and HTLV-1 aB from 2 to 5 sequences. The overall data confirmed that the cosmopolitan transcontinental sub-subtypes were the most prevalent in the Brazilian population. It is hoped that this valuable genomic data will add to our current understanding of the evolutionary history of this medically important virus.

## Introduction

Human T-cell leukemia virus type I (HTLV-1) is the retrovirus responsible for adult T-cell leukemia/lymphoma (ATLL) and for the chronic neurological disorder HTLV-1-associated myelopathy/tropical spastic paraparesis (HAM/TSP) [1], [2], [3], [4]. The virus has also been implicated in a variety of inflammatory diseases, such as uveitis [5], pulmonary alveolitis [6], Hashimoto thyroiditis [7], and chronic arthropathy [8]. Globally, an estimated 10–20 million individuals are HTLV-1 carriers [9]. The disease burden is unevenly distributed in endemic areas, particularly in southwest Japan, the Caribbean islands, South America, and portions of Central Africa [10], [11]. Among the 15 to 25 million HTLV-1-infected individuals living throughout the world, approximately 1 to 5% will develop ATL or HAM/TSP, depending on as-yet-unknown cofactors that could vary according to geographical location [12].

Similar to other retroviruses, HTLV-1 carries a diploid RNA genome comprising 9032 nucleotides that is reverse-transcribed into double-stranded DNA that integrates into the host genome as a provirus [13]. This genome contains *gag*, *pol* and *env* genes flanked by long terminal repeat (LTR) sequences at both the 5′ and 3′ ends. A distinct molecular structure, known as the *pX* region, that is not present in other retroviruses is found between *env* and the 3′ LTR. The plus strand of the *pX* region encodes the regulatory proteins p40 (Tax), p27 (Rex), p12, p13, p30, and p21, which are critical to the viral infectivity in resting primary lymphocytes and to proliferation in infected cells [14].

Much of our current understanding of the HTLV-1 genome structure, variability and evolution has come from the conventional Sanger di-deoxy sequencing approach applied to viral partial sequences. According to previously published data on phylogenetic comparisons of partial sequences, seven subtypes of HTLV-1 strains have been described thus far (a–g) [15]: the cosmopolitan subtype A, the Australo-Melanesian subtype C and the Central African subtype B, D, E, F and G. The cosmopolitan subtype A is further divided into five sub-subtypes : (A) Transcontinental, (B) Japanese, (C) West African, (D) North African, and (E) the Peruvian Black [11], [16], [17]. However, only 2 HTLV-1 subtypes (a and 1b) have had their whole genomes sequenced to date. The data on the complete genome sequences of the HTLV-1 strains found in Brazil are scant. Of note, HTLV-1 infection is endemic in Brazil, and the prevalence varies across different regions of Brazil [18], [19]. Recently, it has been reported that the overall seroprevalence of HTLV infection among 281,760 first-time donors from three blood centers in Brazil was approximately 135 per 10^5^ [19]. The same study reported an incidence of 3.6×10^5^ person-years, and the residual transfusion risk was 5.0×10^6^ per blood unit transfused. A high prevalence of HTLV-1 infection has been reported in Salvador, a large city in the eastern part of Brazil, with an estimated prevalence of 1.35% among blood donors and 1.76% of the overall population [20]. The transcontinental sub-subtypes found in Brazil are believed to have been recently introduced from Africa, most likely through the post-Columbian migrations of the African slave trade between the sixteenth and nineteenth centuries[20], [21].

DNA sequencing has been dramatically advanced by increasingly high-throughput technology. Recent work has employed this technology to enable the characterization of entire viral populations in human and nonhuman primates [22], [23], [24] and to identify minor genomic variants [25], [26]. It is somewhat surprising then that despite millions of people being infected with HTLV-1 worldwide, what was known about HTLV-1 strain genomes was primarily derived from shorter partial sequences of the viral genomes. The scarcity of HTLV-1 complete genome sequences prompted us to characterize and generate newer genetic materials of these viruses, which provide a useful tool for studying viral origin and evolution, in addition to aiding epidemiological monitoring. Here, we combined Illumina's sequencing by synthesis (SBS) technology with a transposon-based fragmentation method to perform genome wide ultra-deep sequencing of 90 HTLV-1 amplified genomes. From this data, we sought to investigate whether different molecular subtypes were associated with disease development in the participating subjects.

## Materials and Methods

### Study Population

Ninety participants were randomly selected from a larger cohort of 233 HTLV-1-infected persons representing 48 (53.3%) asymptomatic carriers (ACs), 35 (38.9%) HAM/TSP patients and 7 (7.8%) ATLL patients. This sub-cohort was part of an ongoing project to profile human T-cells miRNAs in the course of HTLV-1 infection using a deep-sequencing approach. A decision to include 90 samples was made because up to 96 samples can be pooled and sequenced together in a single flow cell. HTLV-1-positive individuals were recruited from the HTLV-1 outpatient clinic at the University of Sao Paulo and the Institute of Infectious Diseases “Emilio Ribas.” All ACs were diagnosed as HTLV-1 carriers at the time of blood donation. Viral infection was identified by the Murex HTLV I + II (Abbott/Murex, Wiesbaden, Germany) and Vironostika HTLVI/II (bioMérieux bv, Boxtel, Netherlands) HTLV enzyme immunoassays, and infection was confirmed by HTLV BLOT 2.4 (HTLV blot 2.4, Genelabs Diagnostics, Science Park, Singapore). The clinical status of HAM/TSP was determined based on the WHO criteria for HTLV-1-associated diseases [27]. Diagnostic criteria for ATLL included serologic evidence of HTLV-1 infection and cytologically or histologically proven T cell malignancy. Written informed consent was obtained from each participant. The study was approved by the local review board (Comissão de Ética para Análise de Projetos de Pesquisa, CAPPesq).

### DNA extraction and HTLV-1 proviral load determination

DNA was extracted from peripheral blood mononuclear cells (PBMCs) using a commercial kit (Qiagen GmbH, Hilden Germany) following the manufacturer's instructions. The extracted DNA was used as a template to amplify a 97-bp fragment from the HTLV-1 *tax* region using previously published primers [28]. The TaqMan real-time PCR assay was conducted in a 25-µL reaction mixture containing 10 µL of KAPA PROBE FAST Universal qPCR Master mix kit (KapaBiosystems), 5 µL of template DNA, 0.4 µM of each primer and 0.2 µM of the final concentration of each probe. Amplification and analysis were performed with the Applied Biosystems 7500 real-time PCR system using an initial denaturation step at 95°C for 2 minutes, followed by 40 cycles of 95°C for 10 seconds and 57°C for 45 seconds. A fragment of the *RNase P* gene from humans [29] was used as an internal control. A negative, no-template control (H_2_O control) was run with every assay. Standard curves for HTLV-1 *tax* were generated from MT-2 cells of log_10_ dilutions (from 10^5^ to 10^0^ copies). The threshold cycle for each clinical sample was calculated by defining the point at which the fluorescence exceeded a threshold limit. Each sample was assayed in duplicate, and the mean of the two values was considered the copy number of the sample. The HTLV-1 proviral load was calculated as the copy number of HTLV-1 (*tax*) per 1000 cells = (copy number of HTLV-1 *tax*)/(copy number of *RNase P* gene/2)×1000 cells. The method could detect 1 copy per 10^3^ PBMCs.

### Amplification of the complete provirus genomes

The complete provirus genome was amplified in two large fragments (A, 4.939 bp, nt 10 to 4930, and B, 4562 bp, nt 4459 to 9006) from 200–300 ng of extracted genomic DNA. The structural and regulatory genes of each sequence were mapped based on comparison with the genomic sequence of B1033-2009 sub-subtype aB from Japan (GenBank accession no. AB513134). Fragment A was amplified using the primers HTLV-1 FG_O1S 5′**TGA CAA TGA** CCA TGA GCC CCA AAT ATC CCC CGG′3 and HTLV-1 FG_O1R 5′**GCT GGA GTC** GGG GGG AGT GGT GAA GCT GCC′3. Fragment B was amplified using the primers HTLV-1 FG_O2S 5′**CGC CGG** GGC CTA CTT CCT AAC CAC ATC TGG CAA GG′3 and HTLV-1 FG_O2R 5′**TCT CCT GAG AGT GCT ATA** GGA TGG GCT GTC GCT GGC TCC TAT′3. The boldface nucleotides (non-HTLV-1 specific sequences) are tails at the 5′ end of the outer primers and were added to enhance the nested amplification with inner primers. The PCR products were then subjected to nested PCR to amplify the A and B nested fragments. The nested primers for fragment A were HTLV-1 FG_N1S 5′CCA TGA GCC CCA AAT ATC CCC CGG′3 and HTLV-1 FG_N1R 5′GGG GGG AGT GGT GAA GCT GCC′3. The nested primers for fragment B were HTLV-1 FG_N2S 5′GGC CTA CTT CCT AAC CAC ATC TGG CAA GG′3 and HTLV-1 FG_N2R 5′GGA GCC AGC GAC AGC CCA TCC TAT′3. The PCR conditions for outer and inner PCR were as follows: an initial step of 5 min at 94°C; 35 cycles, with 1 cycle consisting of 30 s at 94°C, 30 s at 60°C, and 5 min at 72°C; and a final step of 10 min at 72°C. The amplified DNA fragments from the nested PCR product were separated by gel electrophoresis and purified using Freeze ‘N Squeeze DNA Gel Extraction Spin Columns. Each purified amplicon was quantified using Quant-IT HS reagents (Invitrogen, Life Technologies, Carlsbad, CA), and both amplicons from a single viral genome were pooled together at equimolar ratios.

### Whole viral genome library preparation

Each pool was then quantitated, and approximately 1 ng of each was used in a fragmentation reaction mix using a Nextera XT DNA sample prep kit according to the manufacturer's protocol. Briefly, tagmentation and fragmentation of each pool were simultaneously performed by incubation for 5 min at 55°C followed by incubation in neutralizing tagment buffer for 5 min at room temperature. After neutralization of the fragmented DNA, a light 12-cycle PCR was performed with Illumina Ready Mix to add Illumina flowcell adaptors, indexes and common adapters for subsequent cluster generation and sequencing. Amplified DNA was then purified using Agencourt AMPure XP beads (Beckman Coulter), which excluded very short library fragments. Following AMPure purification, the quantity of each library was normalized to ensure equally library representation in our pooled samples. Prior to cluster generation, normalized libraries were further quantified by qPCR using the SYBR fast Illumina library quantification kit (KAPA Biosystems) following the instructions of the manufacturer. The qPCR was run on the 7500 Fast Real-Time PCR System (Applied Biosystems). The thermocycling conditions consisted of an initial denaturation step at 95°C for 5 min followed by 35 cycles of [30 s at 95°C and 45 s at 60°C]. The final libraries were pooled at equimolar concentration and diluted to 4 nM. To denature the indexed DNA, 5 µL of the 4 nM library were mixed with 5 µL of 0.2 N fresh NaOH and incubated for 5 min at room temperature. 990 µL of chilled Illumina HT1 buffer was added to the denatured DNA and mixed to make a 20 pM library. After this step, 360 µL of the 20 pM library was multiplexed with 6 µL of 12.5 pM denatured PhiX control to increase sequence diversity and then mixed with 234 µL of chilled HT1 buffer to make a 12 pM sequenceable library. Finally, 600 µL of the prepared library was loaded on an Illumina MiSeq clamshell style cartridge for paired end 250 sequencing.

### Data analysis

Fastq files were generated by the Illumina MiSeq reporter for downstream analysis and validated to evaluate the distribution of quality scores and to ensure that quality scores do not drastically drop over each read. Validated fastq files from each viral genome were *de novo* assembled into contiguous sequences and annotated with CLC Genomics Workbench version 5.5 (CLC Bio, Aarhus, Denmark) and the Sequencher program 5.2 (Gene Code Corp., Ann Arbor, MI). To improve the quality of reads, approximately 10 nucleotides were trimmed from the 3′ end of the reads from each sequence library of each sample. Because the repetitive and identical sequences of both LTR presented the biggest difficulty in assembling HTLV-1 genome sequence data, we decided to only use the 3′ LTR sequence from each sequence for further analysis. The contiguous genomic sequence from each virus strain was extracted from the assembly and used for further analysis. The full designation of samples is 0YYBR_CLNXXX, where 0YY stands for the year of study, BR for Brazil, CLN for clinical status, and XXX for the enrolment number.

To increase the reliability of the observed DNA variations, the background error rate was set at 1% meaning that DNA variations detected in at least 1% of the viral sequences within a given sample are genuine variations.

Open reading frames were individually aligned with prototype variant B1033-2009 using the MAFFT algorithm [30]. Individual open reading frame alignments were then concatenated, and the nucleotide-level similarities of the resulting full-length coding genomes (*gag*, *pol*, *env p30* and *tax*) were calculated using MEGA5 [31]. Bayesian inference (BI) phylogenetic analyses were conducted using MrBayes v. 3.2 [32]. The settings used for the analysis of the resulting partial and full-length coding genomes were nst = 6, with the gamma-distributed rate variation across sites and a proportion of invariable sites (rates = invgamma). Posterior probability distributions were generated using the Markov Chain Monte Carlo (MCMC) method with four chains being run simultaneously for 1,000,000 generations. Burn-in fraction was set at 2500 and trees were sampled every 100 generations. Due to the large size of the LTR dataset and the limited computer capacity, analyses were run until the average standard deviation of split frequencies fell below 0.05. At termination, parameters and trees were summarized with a burnin of 25%. The plot of log-likelihood values over generations were assessed for adequate sampling and potential scale reduction factors for convergence. All trees were displayed using either FigTree v1.4 (http://tree.bio.ed.ac.uk/) or the freely available Archaeopteryx Java software [33]. Nucleotide similarities were estimated using a maximum composite likelihood model implemented in MEGA version 5.0 software.

### GenBank accession numbers

All consensus genome assemblies generated in this study were submitted to NCBI's GenBank database (Accession numbers KF797891–KF797895, KF797896–KF797912, and KF797896–KF797912).

## Results

In total, 90 blood samples from HTLV-1 infected individuals were included in the study. The participants' ages ranged between 31 and 81 years, and the median age was 56 years. Females constituted 68.9% (n = 62) of the study group. The median measurements of CD4 and CD8 lymphocyte percentage by flow cytometry (FACScan, Becton-Dickinson, Cowley, Oxford) were 45% and 22%, respectively, in 31% of subjects. The median proviral loads defined in this study in all the ACS, HAM/TSP and ATLL groups were 431 copies per 10^3^ PBMCs (range, 2–420), 177 copies per 10^3^ PBMCs (range, 4–1035) and 273 copies per 10^3^ PBMCs (153–3279), respectively. The mean time to HTLV-1 diagnosis was 8.8±5.4 years. The characteristics of the 90 patients included in the study are summarized in **Table 1**.

**Table 1:** Demographic and Clinical Characteristics of the HTLV-1 Patients (n = 90).

**Age (years)**	
Mean ±SD	55.9±10.6
Median	56
Range	31–81
**Gender (%)**	
Male	62 (68.9)
Female	28 (31.1)
**Clinical status (%)**	
Asymptomatic carriers (ACs)	48(53.3)
HAM/TSP	35(38.9)
ATLL	7(7.8)
1 **Median lymphocyte subpopulations**	
% CD4 cells	45
% CD8 cells	22
2 **Median proviral load/1000 PBMCs**	
Asymptomatic carriers	43 (2–420)
HAM/TSP	177 (4–1035)
ATLL	224 (153–3297)
**Mean follow-up after HTLV-1 diagnosis (years)**	8.8±5.4

1 The median CD4 and CD8 percentage were measured in 31% of subjects (n = 28).

2 Patients with ATLL or HAM/TSP had significantly higher median proviral loads than ACs (P<0.001), while no significant difference was observed between the ATLL and HAM/TSP groups P = 0.06 (Mann-Whitney test).

**Figure 1:**
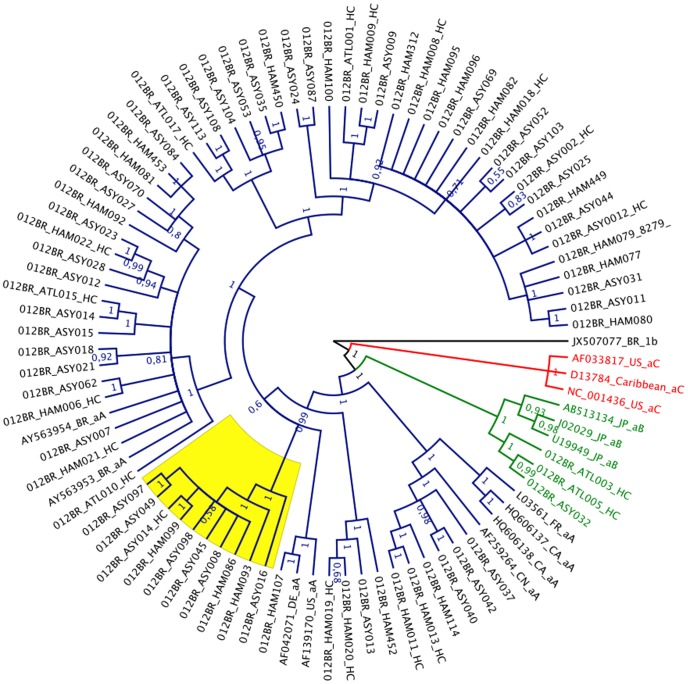
Phylogenetic tree of HTLV-1 sub-subtypes based on Bayesian Inference analysis of the complete coding region sequences (7593 bp, nucleotide 804–8397 according to position in B1033-2009 “GenBank accession no. AB513134”) of 76 participant samples. Colored (blue, sub-subtype aA; red, sub-subtype aB) and black branches represent patient samples and reference sequences from all verified sub-subtypes, respectively. Sequences displayed simultaneous base substitutions over the complete coding region (see Table 2) and formed a monophyletic cluster are indicated by yellow box. For clarity, the tree was midpoint rooted. Values at the nodes represent Bayesian probabilities.

PCR amplifications were successful in all samples, even those harboring proviral copy numbers as low as 2 copies per 10^3^ PBMCs. To assess the genetic variability of HTLV-1, the FLG sequences were successfully assembled into a single, high-quality contig in 76 HTLV-1 sequences, with the majority starting approximately at position 727 of the 5′ long terminal repeat (LTR) to the extreme 3′ LTR at position 9113 (average length of 8259 bp). No ambiguities were detected between the two overlapping sequences, indicating that errors due to PCR had little effect on the overall sequences of the proviral genome of these sequences. The read-through translation of the 5 open reading frames indicated that all complete coding sequences obtained in this study were intact and in frame. There were several nucleotide substitutions in the complete coding regions. Among these, we found 29 specific nucleotides that were always simultaneously substituted in 27 participants and were identical to the prototype Japanese ATK (sub-subtype aB, GenBank accession number J02029) (**Table 2**). Almost all of these substitutions were detected in 13 of 43 asymptomatic carriers, 12 of 31 HAM/TSP patients and 2 of 6 patients with ATLL. In 10 cases with such nucleotide substitutions, several other base substitutions were always simultaneously observed (**Table 2**). Close inspection of the BI tree inferred from the complete coding region alignment indicated that the viral sequences from these 10 cases clustered closely, forming a monophyletic lineage that falls into the transcontinental sub-subtype A cluster (**Figure 1**, highlighted in yellow). These results clearly indicate that variations in HTLV-1 are not randomly distributed but seem to be arranged in hot spots.

**Table 2:** Alignment of nucleotide variations detected simultaneously in the HTLV-1 proviral complete coding region from 27 participants.

	1028	1108	1148	1382	2121	2168	2503	2814	2844	2937	2940	2991	3249	3489	3519	3612	3920	4130	4182	4260	4725	4749	4783	4866	5191	5602	5695	6376	6593	6732	6793	6907	6923	7003	7009	7093	7147	7183	7242	7254	7367	7880	7940	8261	8279	8357
ATK J02029	G	C	C	T	C	G	A	G	C	A	A	C	C	T	A	A	G	G	G	C	C	T	T	T	G	T	T	G	T	T	C	G	C	T	T	T	T	G	A	G	C	T	C	G	T	G
012BR_ATL003_HC	.	.	.	.	.	.	.	.	.	.	.	.	.	.	.	.	.	.	.	.	.	.	.	.	.	.	.	.	.	.	.	.	.	.	.	.	.	.	.	.	.	.	.	.	.	.
012BR_ATL005_HC	.	.	.	.	.	.	.	.	.	.	.	.	.	.	.	.	.	.	.	.	.	.	.	.	.	.	.	.	.	.	.	.	.	.	.	.	.	.	.	.	.	.	.	.	.	.
012BR_HAM011_HC	.	.	.	.	.	.	.	.	.	.	.	.	.	.	.	g	.	.	.	.	.	.	.	.	.	.	.	.	.	.	.	.	.	c	.	.	.	.	.	.	.	.	.	.	.	.
012BR_HAM013_HC	.	.	.	.	.	.	.	.	.	.	.	.	.	.	.	g	.	.	.	.	.	.	.	.	.	.	.	.	.	.	.	.	.	c	.	.	.	.	.	.	.	.	.	.	.	.
012BR_ASY013	.	.	.	.	.	.	.	.	.	.	.	.	.	.	.	.	.	.	.	.	.	.	.	.	.	.	.	.	.	.	.	.	.	.	.	.	.	.	.	.	.	.	.	.	.	.
012BR_HAM114	.	.	.	.	.	.	.	.	t	.	.	.	.	.	.	.	.	.	.	.	.	.	.	.	.	.	.	.	.	.	.	.	.	.	.	.	.	.	.	.	.	.	.	.	.	.
012BR_ASY016	.	.	.	.	.	.	.	.	.	g	.	t	.	.	.	.	.	.	.	.	.	c	c	.	a	.	.	.	.	.	.	a	t	.	.	.	.	a	.	.	.	.	.	.	.	.
012BR_HAM019_HC	.	.	.	.	.	.	.	.	.	.	.	.	.	.	.	.	.	.	.	.	.	.	.	.	.	.	.	.	.	.	.	.	.	.	.	.	.	.	.	.	.	.	.	.	.	.
012BR_HAM020_HC	.	.	.	.	.	.	.	.	.	.	.	.	.	.	.	.	.	.	.	.	.	.	.	.	.	.	.	.	.	.	.	.	.	.	.	.	.	.	.	.	.	.	.	.	.	.
012BR_ASY032	.	.	.	.	.	.	.	.	.	.	.	.	.	.	.	.	a	.	.	.	.	.	.	.	.	.	.	.	.	.	.	.	.	.	.	.	.	.	.	.	.	.	.	.	.	.
012BR_ASY036	-	-	-	-	.	.	.	.	.	g	.	.	.	.	.	.	.	.	.	.	.	.	.	.	.	.	.	.	.	.	.	.	.	.	.	.	.	.	.	.	.	.	.	.	.	.
012BR_ASY037	.	.	.	.	.	.	.	.	.	.	.	.	.	.	.	.	.	.	.	.	.	.	.	.	.	.	.	.	.	.	.	.	.	.	.	.	.	.	.	.	.	.	.	.	.	.
012BR_ASY040	.	.	.	.	.	.	.	.	.	.	.	.	.	.	.	.	.	.	.	.	.	.	.	.	.	.	.	.	.	.	.	.	.	.	.	.	.	.	.	.	.	.	.	.	.	.
012BR_ASY042	.	.	.	.	.	.	.	.	.	.	.	.	.	.	.	.	.	.	.	.	.	.	.	.	.	.	.	.	.	.	.	.	.	.	.	.	.	.	.	.	.	.	.	.	.	.
012BR_HAM107	.	.	.	.	.	.	.	.	.	.	.	.	.	.	.	.	.	.	.	.	.	g	.	.	.	.	.	.	.	.	.	.	.	.	.	.	.	.	.	.	.	.	.	.	.	.
012BR_HAM426	-	-	-	-	-	-	-	.	.	.	.	.	.	.	.	.	.	.	.	.	.	.	.	.	.	.	.	.	.	.	.	.	.	.	.	.	.	.	.	.	.	.	.	.	.	.
012BR_HAM452	.	.	.	.	.	.	.	.	.	.	.	.	.	.	.	.	.	.	.	.	.	.	.	.	.	.	.	.	.	.	.	.	.	.	.	.	.	.	.	.	.	.	.	.	.	.
*012BR_ASY008*	a	t	t	.	.	.	.	.	.	g	.	t	.	.	.	.	.	a	.	t	.	c	c	c	a	.	.	.	.	.	.	a	t	c	.	.	.	a	.	.	.	.	.	a	.	a
*012BR_ASY014_HC*	a	t	t	.	.	.	.	.	.	g	.	t	.	.	.	.	.	a	.	t	.	c	c	c	a	.	.	.	.	.	.	a	t	c	.	.	.	a	.	.	.	.	.	a	.	a
*012BR_HAM016_HC*	-	t	t	.	.	.	.	.	.	g	.	t	.	.	.	.	.	a	.	t	.	c	c	c	a	.	.	.	.	.	.	a	t	c	.	.	.	a	.	.	.	.	.	-	-	-
*012BR_ASY045*	a	t	t	.	.	.	.	.	.	g	.	t	.	.	.	.	.	a	.	t	.	c	c	c	a	.	.	.	.	.	.	a	t	c	.	.	.	a	.	.	.	.	.	a	.	a
*012BR_ASY049*	a	t	t	.	.	.	.	.	.	g	.	t	.	.	.	.	.	a	.	t	.	c	c	c	a	.	.	.	.	.	.	a	t	c	.	.	.	a	.	.	.	.	.	a	.	a
*012BR_HAM086*	a	t	t	.	.	.	.	.	.	g	.	t	.	.	.	.	.	a	.	t	.	c	c	c	a	.	.	.	.	.	.	a	t	c	.	.	.	a	.	.	.	.	.	a	.	a
*012BR_HAM093*	a	t	t	.	.	.	.	.	.	g	.	t	.	.	.	.	.	a	.	t	.	c	c	c	a	.	.	a	.	.	.	a	t	t	.	.	.	a	.	.	.	.	.	a	.	a
*012BR_ASY097*	a	t	t	.	.	.	.	.	.	g	.	t	.	.	.	.	.	a	.	t	.	c	c	c	a	.	.	.	.	.	.	a	t	c	.	.	.	a	.	.	.	.	.	a	.	a
*012BR_ASY098*	a	t	t	.	.	.	.	.	.	g	.	t	.	.	.	.	.	a	.	t	.	c	c	c	-	.	.	a	.	.	.	a	t	c	.	.	.	a	.	.	.	.	.	a	.	a
*012BR_HAM099*	a	t	t	.	.	.	.	.	.	g	.	t	.	.	.	.	.	a	.	t	.	c	c	c	a	.	.	.	.	.	.	a	t	c	.	.	.	a	.	.	.	.	.	a	.	a
AY563953_aA	.	.	.	c	a	t	t	a	t	g	g	.	t	c	g	c	a	.	a	.	t	.	.	.	.	g	c	a	c	g	t	.	.	.	c	c	c	.	g	a	t	c	t	.	c	.

Dashes indicate gaps; dots indicate identity with the prototype Japanese ATK (sub-subtype aB, GenBank accession number J02029). Nucleotide substitutions are enclosed in boxes. Sequences written in italics displayed additional simultaneous base substitutions.

The BI tree analysis from the complete coding region determined that HTLV-1 strains from our patients belonged to the cosmopolitan transcontinental sub-subtypes or HTLV-1 aA, except for 3 (4%) samples (012BR ATL003 HC, 012BR ASY032, and 012BR ATL005 HC), which belonged to the Japanese HTLV-1 aB sub-subtypes and are represented in this tree by a single branch (**Figure 1**). The HTLV-1 aA was detected in all patients with HAM/TSP, 40 of 41 ASCs, and 4 of 6 patients with ATLL. No distinctive mutations were observed among the viral sequences from the three clinical groups. Neither the ASCs nor the HAM/TSP or ATLL sequences formed a unique cluster. The maximum nucleotide distances within each group were 0.5%, 0.7% and 0.5% in the TSP/HAM, ATLL and asymptomatic HTLV-1 infected patients, respectively. The complete genomes of HTLV-1 aA had a mean nucleotide divergence from HTLV-1 aB of 1% (**Table 3**). Based on the latter analysis, it was hard to determine the relationship between viral sequence and virulence. Furthermore, HTLV-1 aA had a slightly higher intragroup divergence than sequences belonging to HTLV-1 aB. The sequence analysis of the *tax* gene of the sequences with complete coding regions confirmed the presence of the complete set of four polymorphic sites [34] characteristic of *tax* A in 73 (96%) samples, but the *tax* B profile was confirmed in only 3 (4%) strains, namely 012BR ATL003 HC, 012BR ASY032, and 012BR ATL005 HC, which clustered within the *tax B* sub-subtypes (**Figure 1**).

**Table 3:** Comparison of the nucleotides of the transcontinental (A) and Japanese (B) sub-subtypes of the cosmopolitan genotype*.

	Intragroup (%)		Intergroup (%)
	HTLV-1aA	HTLV-1aB	HTLV-1aA *vs* HTLV-1aB
Complete genome	0.5	0.2	1.0
*gag*	0.3	0.2	0.8
polymerase	0.4	0.3	0.8
envelope	0.5	0.2	1.1
pX	0.9	0.2	1.7

*The intra- and intergroup comparisons were performed with the new sequences generated in this study.

Fourteen HTLV-1 sequences, identified by a phylogenetic tree from partial data as belonging to the HTLV-1 aA (n = 13) or HTLV-1 aB (n = 1) sub-subtypes (**Figure S1**), failed to generate full genomic data by our deep sequencing method. The *de novo* assembly of the compiled genome from each of these sequences had an average sequencing depth ranging from 351–5713. When aligned to the B1033-2009 sequence, eight sequences displayed two contigs separated by a gap of less than 500 bp, suggesting the small size of most gaps (**Figure S1**).

Next, the intra-sample single nucleotide variability within each sample with partial and/or complete coding regions was investigated for potential quasispecies characterized by nucleotide substitutions. Based on our rigid criteria, the presence of genuine DNA variant could not be detected in any of the samples. Hence, it appeared that no minority viral variants were present in the 90 blood samples analyzed by illumina next generation sequencing.

**Figure 2:**
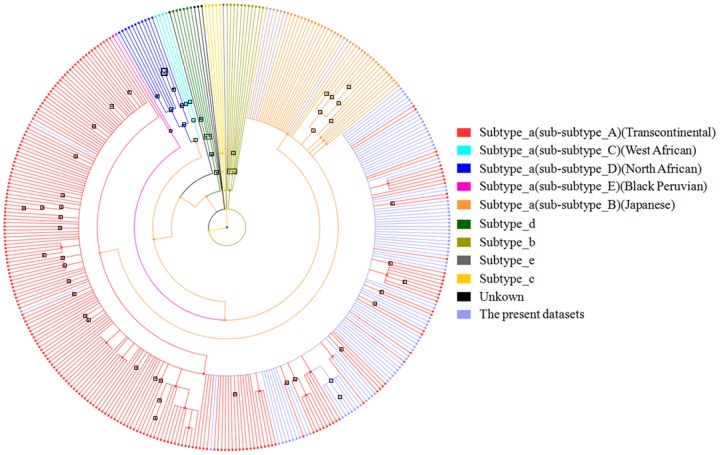
Phylogenetic tree of HTLV-1 sub-subtypes based on Bayesian Inference analysis from the long terminal repeat (LTR, 664 bp) of 88 participant samples and 279 HTLV-1 LTR sequences from the database representing 5 of the HTLV-1 subtypes. The representatives of the 5 references are color-coded. Branches with posterior probabilities ≥0.70 are displayed with blank square.

**Figure 3:**
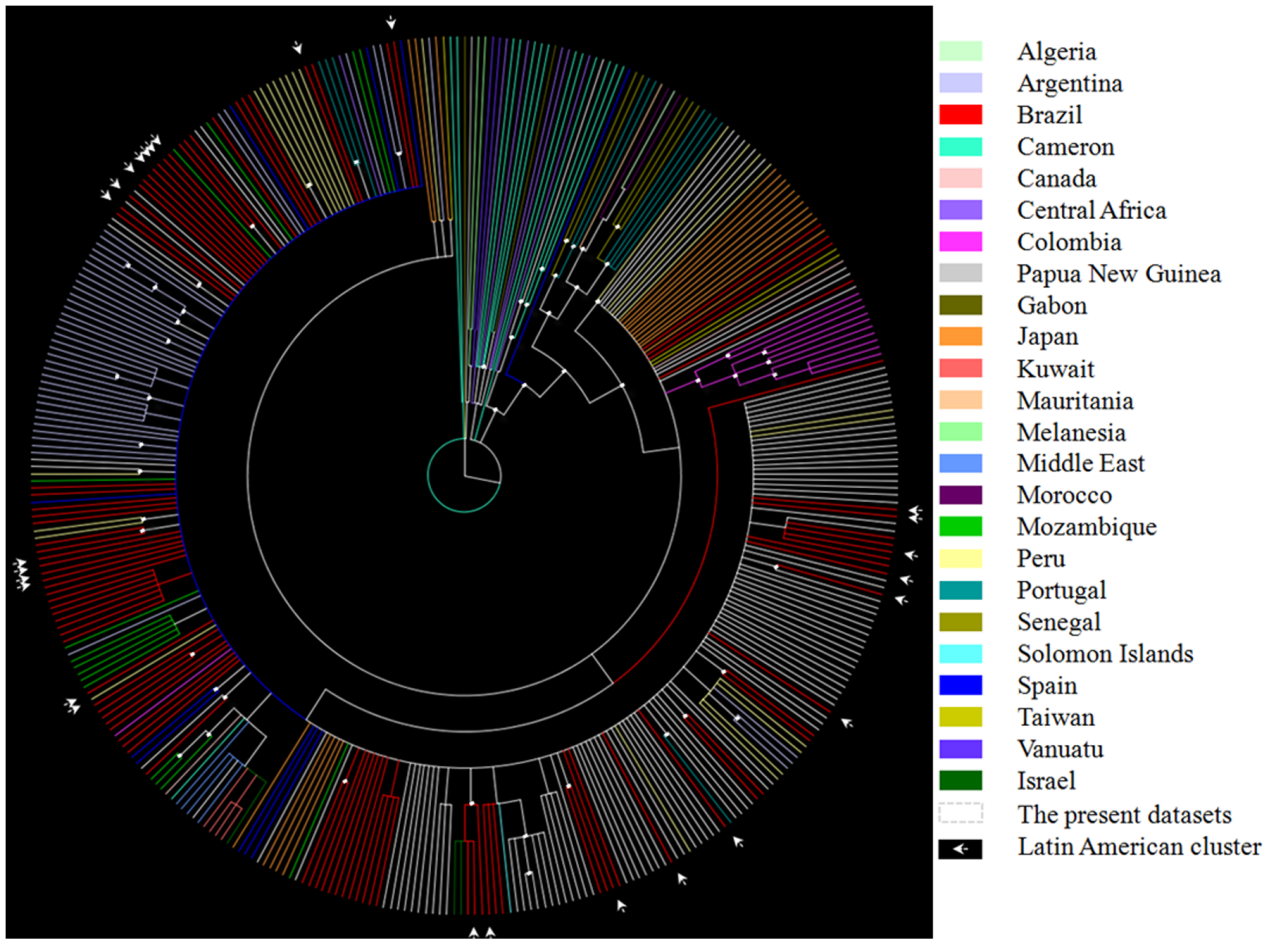
Bayesian analysis of 297 long terminal repeat (LTR) sequences from various global locations (colored branch), including previously published Brazilian and other South American sequences from neighboring countries. The tree also contains 88 sequences from the current study. Branches with posterior probabilities ≥0.70 are displayed (white dots).

To further explore the genetic variation and subtype classifications within the HTLV subtype, all available LTR sequences (n = 88) were extracted from the HTLV complete and partial genomes of each subject and aligned with the LTR sequences representing all assigned subtypes and unassigned variants with a minimum length of 500 bp from the HTLV-1 molecular epidemiology database (http://htlv1db.bahia.fiocruz.br/). On the basis of a phylogenetic analysis of this region (**Figure 2**), 84 subjects were classified as being infected with subtype aA, and 4 individuals were infected with subtype aB. Moreover, all sequences classified as subtype aA or aB on the basis of the complete coding region depicted in **Figure 1** displayed a concordant subtype classification in LTR sub-genomic regions, suggesting the absence of inter-genomic recombination. As shown in **Figure 2**, the LTR sequences from the current study (faint blue branches) were dispersed among other HTLV-1 aA sequences. Moreover, the phylogenies of subtype aA displayed no considerable grouping of sequences by clinical status. To investigate the origin of HTLV-1 subtypes in Brazil, we compiled the LTR data sets of the current study (n = 88 sequences) and all reference sequences (n = 297 sequences) for these subtypes from different geographic origins, including Brazil (**Figure 3**). No significant unique cluster of Brazilian HTLV-1 aA was observed. Instead, these subtypes are all interspersed with strains from different geographic locations, mainly in South America, Europe, Africa and Asia. Seven of the 11 (4 ACs and 3 HAM/TSP) patients of Japanese descent (n = 11) or those who had sexual contact with a Japanese partner (n = 1) in this study were infected with HTLV-1 a grouped in a monophyletic cluster within the Japanese *tax* sequences that belong to the LTR sub-subtypes of cosmopolitan A (**Figure 3**). This group was also separated by an excellent aLRT value (93%) in the complete coding region (**Figure 1**). In the case of subtype bA (**Figure 3**), the 4 LTRs from this study were sequenced from Japanese descendants and positioned with other Brazilian sequences in a main cluster of sequences that originated from Japan, Taiwan, Argentina and Colombia.

## Discussion

Currently, there are only 15 complete sequences of HTLV-1 sequences in the GenBank and HTLV-1 molecular epidemiology databases (http://htlv1db.bahia.fiocruz.br/) that were classified as subtypes aA (n = 8), aB (n = 2), aC (n = 2) and 1b (n = 1) and 2 unassigned, for which some of the sequences have the country of sampling stated. Among these, there were 3 recovered from Brazil. The scarcity of these sequences prompted us to generate newer genetic materials of these viruses and to obtain more information on the molecular basis of HTLV-1 sequences in Brazil. To date, no study has employed second-generation sequencing techniques to examine HTLV-1 heterogeneity across entire genomes from different clinical settings. In this work, we used state-of-the-art Illumina MiSeq instrumentation to generate 76 and 14 HTLV-1 complete and partial genome sequences, respectively, from different ACs, HAM/TSP and ATLL clinical sources in a single run. Thus, the sequences described in this study quintuple the publicly available full genome sequence information for HTLV-1 viruses. Analysis indicates that the vast majority of subjects in this study was infected with the cosmopolitan genotype, mostly the transcontinental and more rarely the Japanese sub-subtypes, a finding that is in agreement with previous reports [35], [36], [37], [38]. Nevertheless, we obtained no evidence that a specific HTLV-1-subtypes or sub-subtypes are associated with a certain clinical status. This finding is in accordance with earlier studies demonstrating that the nucleotide substitutions in some fragments of the HTLV-1 genome were specific for the geographic origin of the patients rather than for the type of associated pathologies [36], [39], [40], [41]. Regardless of the patient's clinical status, sequence homogeneity between strains recovered from a common geographical origin is often seen.

In the present study, 88 Brazilian HTLV-1 LTR sequences were extracted from their complete genomes and used to reconstruct the phylogenetic history of the virus in this country. This region has previously been shown to provide a sufficiently strong phylogenetic signal to allow the distinction of HTLV-1 sub-subtypes and contains a larger number of HTLV-1 sequences in the database [42]. It is important to note that the best resolution of evolutionary patterns is obtained from complete genomes. However, this was not possible because there were only a few HTLV-1 complete genome sequences publicly available. As expected, all of the transcontinental strains identified in this study were clustered in different branches with strains from different geographic origins in Europe, Africa, Asia and, mainly, South America, corresponding to the formerly named Latin American cluster. The grouping of the Brazilian HTLV-1 sequences into different subclusters support the hypothesis that there were multiple introductions of the transcontinental subtype in Brazil. These findings further support several studies conducted in some Brazilian and other Latin American populations that suggested the introduction of HTLV-1 on multiple occasions and that demonstrate an association between the Latin American cluster with sequences of African origin [37], [43], [44], [45], [46]. The results of detecting HTLV-1 aA and aB among patients of Japanese descent is consistent with previous data described among Japanese immigrants in Sao Paulo, where the detection of both sub-subtypes has been reported [17], [35], [47], [48].

In conclusion, we provided new full-length genome sequences of HTLV-1 from the different clinical setting determined by deep sequencing for two different variants. Therefore, this study has increased the number of subtype aA full-length genomes to 81 and HTLV-1 aB to 5 sequences. Together with other partial sequences, we confirmed that HTLV-1 subtype a transcontinental sub-subtypes A was the most prevalent in the Brazilian population. We believe that these data open avenues for further studies on the evolutionary relationships between the HTLV-1 subtypes and may contribute to the information on the genetic diversity of HTLV-1 worldwide.

## Supporting Information

Figure S1 **Schematic representation of the sequences that failed to generate full genomic data when subjected to our deep sequencing method.** Consensus sequence reads were aligned and mapped to the Brazilian reference sequence (GenBank: AY563953.1) to define their genomic locations. The star symbol indicates proviral load (number of proviral copies per 1000 cells). The pilcrow symbol indicates the overall mean coverage depth.
(TIF)

## References

[1] Gessain A, Barin F, Vernant JC, Gout O, Maurs L, et al (1985) Antibodies to human T-lymphotropic virus type-I in patients with tropical spastic paraparesis. Lancet 2: 407–410. 2863442 10.1016/s0140-6736(85)92734-5

[2] Osame M, Usuku K, Izumo S, Ijichi N, Amitani H, et al (1986) HTLV-I associated myelopathy, a new clinical entity. Lancet 1: 1031–1032. 10.1016/s0140-6736(86)91298-5 2871307

[3] Poiesz BJ, Ruscetti FW, Gazdar AF, Bunn PA, Minna JD, et al (1980) Detection and isolation of type C retrovirus particles from fresh and cultured lymphocytes of a patient with cutaneous T-cell lymphoma. Proc Natl Acad Sci U S A 77: 7415–7419. 6261256 10.1073/pnas.77.12.7415 PMC350514

[4] Yoshida M, Miyoshi I, Hinuma Y (1982) Isolation and characterization of retrovirus from cell lines of human adult T-cell leukemia and its implication in the disease. Proc Natl Acad Sci U S A 79: 2031–2035. 6979048 10.1073/pnas.79.6.2031 PMC346116

[5] Huang YQ, Li JJ, Nicolaides A, Zhang WG, Freidman-Kien AE (1992) Fibroblast growth factor 6 gene expression in AIDS-associated Kaposi's sarcoma. Lancet 339: 1110–1111. 10.1016/0140-6736(92)90702-5 1349122

[6] Sugimoto M, Nakashima H, Watanabe S, Uyama E, Tanaka F, et al (1987) T-lymphocyte alveolitis in HTLV-I-associated myelopathy. Lancet 2: 1220. 10.1016/s0140-6736(87)91362-6 2890850

[7] Kawai H, Saito M, Takagi M, Tsuchihashi T, Arii Y, et al (1992) Hashimoto's thyroiditis in HTLV-I carriers. Intern Med 31: 1213–1216. 1286230 10.2169/internalmedicine.31.1213

[8] Nishioka K, Maruyama I, Sato K, Kitajima I, Nakajima Y, et al (1989) Chronic inflammatory arthropathy associated with HTLV-I. Lancet 1: 441. 10.1016/s0140-6736(89)90038-x 2563817

[9] Edlich RF, Arnette JA, Williams FM (2000) Global epidemic of human T-cell lymphotropic virus type-I (HTLV-I). J Emerg Med 18: 109–119. 10645850 10.1016/s0736-4679(99)00173-0

[10] Matsuoka M (2003) Human T-cell leukemia virus type I and adult T-cell leukemia. Oncogene 22: 5131–5140. 12910250 10.1038/sj.onc.1206551

[11] Proietti FA, Carneiro-Proietti AB, Catalan-Soares BC, Murphy EL (2005) Global epidemiology of HTLV-I infection and associated diseases. Oncogene 24: 6058–6068. 16155612 10.1038/sj.onc.1208968

[12] Gessain A, Cassar O (2012) Epidemiological Aspects and World Distribution of HTLV-1 Infection. Front Microbiol 3: 388. 23162541 10.3389/fmicb.2012.00388 PMC3498738

[13] Seiki M, Hattori S, Hirayama Y, Yoshida M (1983) Human adult T-cell leukemia virus: complete nucleotide sequence of the provirus genome integrated in leukemia cell DNA. Proc Natl Acad Sci U S A 80: 3618–3622. 6304725 10.1073/pnas.80.12.3618 PMC394101

[14] Franchini G, Fukumoto R, Fullen JR (2003) T-cell control by human T-cell leukemia/lymphoma virus type 1. Int J Hematol 78: 280–296. 14686485 10.1007/BF02983552

[15] Verdonck K, Gonzalez E, Van Dooren S, Vandamme AM, Vanham G, et al (2007) Human T-lymphotropic virus 1: recent knowledge about an ancient infection. Lancet Infect Dis 7: 266–281. 17376384 10.1016/S1473-3099(07)70081-6

[16] Vidal AU, Gessain A, Yoshida M, Tekaia F, Garin B, et al (1994) Phylogenetic classification of human T cell leukaemia/lymphoma virus type I genotypes in five major molecular and geographical subtypes. J Gen Virol 75 Pt 12: 3655–3666. 7996161 10.1099/0022-1317-75-12-3655

[17] Van Dooren S, Gotuzzo E, Salemi M, Watts D, Audenaert E, et al (1998) Evidence for a post-Columbian introduction of human T-cell lymphotropic virus [type I] [corrected] in Latin America. J Gen Virol 79 Pt 11: 2695–2708. 9820145 10.1099/0022-1317-79-11-2695

[18] Galvao-Castro B, Loures L, Rodriques LG, Sereno A, Ferreira Junior OC, et al (1997) Distribution of human T-lymphotropic virus type I among blood donors: a nationwide Brazilian study. Transfusion 37: 242–243. 9051104 10.1046/j.1537-2995.1997.37297203532.x

[19] Carneiro-Proietti AB, Sabino EC, Leao S, Salles NA, Loureiro P, et al (2012) Human T-lymphotropic virus type 1 and type 2 seroprevalence, incidence, and residual transfusion risk among blood donors in Brazil during 2007-2009. AIDS Res Hum Retroviruses 28: 1265–1272. 22324906 10.1089/aid.2011.0143 PMC3448098

[20] Dourado I, Alcantara LC, Barreto ML, da Gloria Teixeira M, Galvao-Castro B (2003) HTLV-I in the general population of Salvador, Brazil: a city with African ethnic and sociodemographic characteristics. J Acquir Immune Defic Syndr 34: 527–531. 14657765 10.1097/00126334-200312150-00013

[21] Catalan-Soares B, Carneiro-Proietti AB, Proietti FA (2005) Heterogeneous geographic distribution of human T-cell lymphotropic viruses I and II (HTLV-I/II): serological screening prevalence rates in blood donors from large urban areas in Brazil. Cad Saude Publica 21: 926–931. 15868051 10.1590/s0102-311x2005000300027

[22] Bimber BN, Burwitz BJ, O'Connor S, Detmer A, Gostick E, et al (2009) Ultradeep pyrosequencing detects complex patterns of CD8+ T-lymphocyte escape in simian immunodeficiency virus-infected macaques. J Virol 83: 8247–8253. 19515775 10.1128/JVI.00897-09 PMC2715741

[23] Bimber BN, Dudley DM, Lauck M, Becker EA, Chin EN, et al (2010) Whole-genome characterization of human and simian immunodeficiency virus intrahost diversity by ultradeep pyrosequencing. J Virol 84: 12087–12092. 20844037 10.1128/JVI.01378-10 PMC2977871

[24] Willerth SM, Pedro HA, Pachter L, Humeau LM, Arkin AP, et al (2010) Development of a low bias method for characterizing viral populations using next generation sequencing technology. PLoS One 5: e13564. 21042592 10.1371/journal.pone.0013564 PMC2962647

[25] Dudley DM, Chin EN, Bimber BN, Sanabani SS, Tarosso LF, et al (2012) Low-cost ultra-wide genotyping using Roche/454 pyrosequencing for surveillance of HIV drug resistance. PLoS One 7: e36494. 22574170 10.1371/journal.pone.0036494 PMC3344889

[26] Wang C, Mitsuya Y, Gharizadeh B, Ronaghi M, Shafer RW (2007) Characterization of mutation spectra with ultra-deep pyrosequencing: application to HIV-1 drug resistance. Genome Res 17: 1195–1201. 17600086 10.1101/gr.6468307 PMC1933516

[27] Osame M (1990) Review of WHO Kagoshima Meeting and diagnostic guidelines for HAM/TSP.In Human Retrovirology: HTLV. Edited by Blattner W. New York: Raven. 191–197.

[28] Heneine W, Khabbaz RF, Lal RB, Kaplan JE (1992) Sensitive and specific polymerase chain reaction assays for diagnosis of human T-cell lymphotropic virus type I (HTLV-I) and HTLV-II infections in HTLV-I/II-seropositive individuals. J Clin Microbiol 30: 1605–1607. 1624585 10.1128/jcm.30.6.1605-1607.1992 PMC265343

[29] Naderi M, Paryan M, Azadmanesh K, Rafatpanah H, Rezvan H, et al (2012) Design and development of a quantitative real time PCR assay for monitoring of HTLV-1 provirus in whole blood. J Clin Virol 53: 302–307. 22306271 10.1016/j.jcv.2011.12.033

[30] Katoh K, Misawa K, Kuma K, Miyata T (2002) MAFFT: a novel method for rapid multiple sequence alignment based on fast Fourier transform. Nucleic Acids Res 30: 3059–3066. 12136088 10.1093/nar/gkf436 PMC135756

[31] Tamura K, Peterson D, Peterson N, Stecher G, Nei M, et al (2011) MEGA5: molecular evolutionary genetics analysis using maximum likelihood, evolutionary distance, and maximum parsimony methods. Mol Biol Evol 28: 2731–2739. 21546353 10.1093/molbev/msr121 PMC3203626

[32] Ronquist F, Teslenko M, van der Mark P, Ayres DL, Darling A, et al (2012) MrBayes 3.2: efficient Bayesian phylogenetic inference and model choice across a large model space. Syst Biol 61: 539–542. 22357727 10.1093/sysbio/sys029 PMC3329765

[33] Han MV, Zmasek CM (2009) phyloXML: XML for evolutionary biology and comparative genomics. BMC Bioinformatics 10: 356. 19860910 10.1186/1471-2105-10-356 PMC2774328

[34] Furukawa Y, Yamashita M, Usuku K, Izumo S, Nakagawa M, et al (2000) Phylogenetic subgroups of human T cell lymphotropic virus (HTLV) type I in the tax gene and their association with different risks for HTLV-I-associated myelopathy/tropical spastic paraparesis. J Infect Dis 182: 1343–1349. 11010842 10.1086/315897

[35] Kashima S, Alcantara LC, Takayanagui OM, Cunha MA, Castro BG, et al (2006) Distribution of human T cell lymphotropic virus type 1 (HTLV-1) subtypes in Brazil: genetic characterization of LTR and tax region. AIDS Res Hum Retroviruses 22: 953–959. 17067264 10.1089/aid.2006.22.953

[36] Segurado AA, Biasutti C, Zeigler R, Rodrigues C, Damas CD, et al (2002) Identification of human T-lymphotropic virus type I (HTLV-I) subtypes using restricted fragment length polymorphism in a cohort of asymptomatic carriers and patients with HTLV-I-associated myelopathy/tropical spastic paraparesis from Sao Paulo, Brazil. Mem Inst Oswaldo Cruz 97: 329–333. 12048560 10.1590/s0074-02762002000300009

[37] Magri MC, Brigido LF, Rodrigues R, Morimoto HK, Ferreira JL, et al (2012) Phylogenetic and similarity analysis of HTLV-1 isolates from HIV-coinfected patients from the south and southeast regions of Brazil. AIDS Res Hum Retroviruses 28: 110–114. 21591992 10.1089/aid.2011.0117 PMC3252815

[38] Neto WK, Da-Costa AC, de Oliveira AC, Martinez VP, Nukui Y, et al (2011) Correlation between LTR point mutations and proviral load levels among human T cell lymphotropic virus type 1 (HTLV-1) asymptomatic carriers. Virol J 8: 535. 22166003 10.1186/1743-422X-8-535 PMC3287369

[39] Leitner T (2002) The molecular epidemiology of human viruses. Berlin, Germany: Springer Science and Business Media.

[40] Komurian F, Pelloquin F, de The G (1991) In vivo genomic variability of human T-cell leukemia virus type I depends more upon geography than upon pathologies. J Virol 65: 3770–3778. 2041093 10.1128/jvi.65.7.3770-3778.1991 PMC241407

[41] Longo DL, Gelmann EP, Cossman J, Young RA, Gallo RC, et al (1984) Isolation of HTLV-transformed B-lymphocyte clone from a patient with HTLV-associated adult T-cell leukaemia. Nature 310: 505–506. 6087161 10.1038/310505a0

[42] Mahieux R, Ibrahim F, Mauclere P, Herve V, Michel P, et al (1997) Molecular epidemiology of 58 new African human T-cell leukemia virus type 1 (HTLV-1) strains: identification of a new and distinct HTLV-1 molecular subtype in Central Africa and in Pygmies. J Virol 71: 1317–1333. 8995656 10.1128/jvi.71.2.1317-1333.1997 PMC191187

[43] Alcantara LC, de Oliveira T, Gordon M, Pybus O, Mascarenhas RE, et al (2006) Tracing the origin of Brazilian HTLV-1 as determined by analysis of host and viral genes. AIDS 20: 780–782. 16514313 10.1097/01.aids.0000216383.14808.13

[44] Mota AC, Van Dooren S, Fernandes FM, Pereira SA, Queiroz AT, et al (2007) The close relationship between South African and Latin American HTLV type 1 strains corroborated in a molecular epidemiological study of the HTLV type 1 isolates from a blood donor cohort. AIDS Res Hum Retroviruses 23: 503–507. 17506606 10.1089/aid.2006.0203

[45] Eirin ME, Dilernia DA, Berini CA, Jones LR, Pando MA, et al (2008) Divergent strains of human T-lymphotropic virus type 1 (HTLV-1) within the Cosmopolitan subtype in Argentina. AIDS Res Hum Retroviruses 24: 1237–1244. 18834325 10.1089/aid.2008.0024

[46] Balcazar N, Sanchez GI, Garcia-Vallejo F (2003) Sequence and phylogenetic analysis of human T cell lymphotropic virus type 1 from Tumaco, Colombia. Mem Inst Oswaldo Cruz 98: 641–648. 12973531 10.1590/s0074-02762003000500010

[47] Song KJ, Nerurkar VR, Pereira-Cortez AJ, Yamamoto M, Taguchi H, et al (1995) Sequence and phylogenetic analyses of human T cell lymphotropic virus type 1 from a Brazilian woman with adult T cell leukemia: comparison with virus strains from South America and the Caribbean basin. Am J Trop Med Hyg 52: 101–108. 7856818 10.4269/ajtmh.1995.52.101

[48] Yamashita M, Ishida T, Ohkura S, Miura T, Hayami M (2001) Phylogenetic characterization of a new HTLV type 1 from the Ainu in Japan. AIDS Res Hum Retroviruses 17: 783–787. 11429119 10.1089/088922201750237068

