# Correction: Molecular Characterization of Human T-Cell Lymphotropic Virus Type 1 Full and Partial Genomes by Illumina Massively Parallel Sequencing Technology

**DOI:** 10.1371/journal.pone.0102223

**Published:** 2014-07-09

**Authors:** The PLOS ONE Staff

The fifth author's name is spelled incorrectly. The correct name is: Jorge Casseb. The correct citation is: Pessôa R, Watanabe JT, Nukui Y, Pereira J, Casseb J, et al. (2014) Molecular Characterization of Human T-Cell Lymphotropic Virus Type 1 Full and Partial Genomes by Illumina Massively Parallel Sequencing Technology. PLoS ONE 9(3): e93374. doi:10.1371/journal.pone.0093374

The affiliation for the fifth author is incorrect. Jorge Casseb is not affiliated with #3, but with the Institute of Tropical Medicine of São Paulo, University of São Paulo, São Paulo, Brazil.
