# Supplementary figures and images for: Molecular Characterization of Human T-Cell Lymphotropic Virus Type 1 Full and Partial Genomes by Illumina Massively Parallel Sequencing Technology
Source: PLoS One. 2014 Mar 31;9(3):e93374. doi: 10.1371/journal.pone.0093374 (PMC3970957; doi:10.1371/journal.pone.0093374)

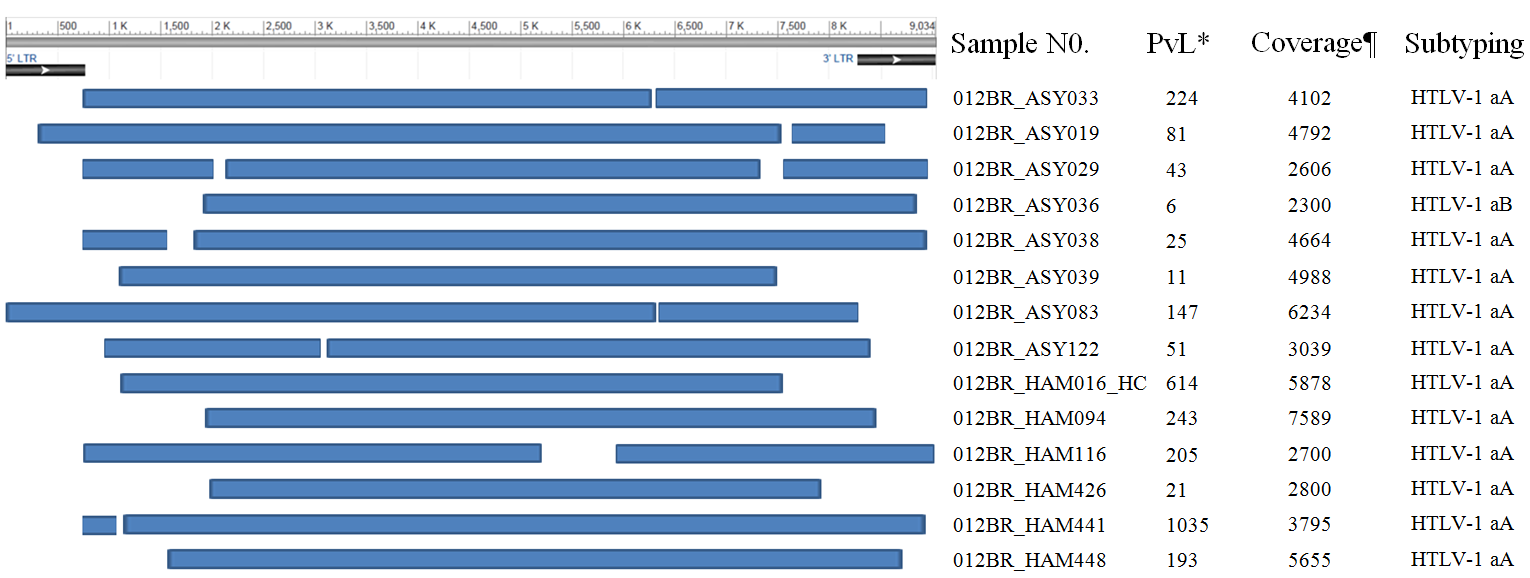

Supplement: Figure S1 — Schematic representation of the sequences that failed to generate full genomic data when subjected to our deep sequencing method. Consensus sequence reads were aligned and mapped to the Brazilian reference sequence (GenBank: AY563953.1) to define their genomic locations. The star symbol indicates proviral load (number of proviral copies per 1000 cells). The pilcrow symbol indicates the overall mean coverage depth. (TIF) [file pone.0093374.s001.tif]
